# Meta-analysis of the Effects of Insect Vector Saliva on Host Immune Responses and Infection of Vector-Transmitted Pathogens: A Focus on Leishmaniasis

**DOI:** 10.1371/journal.pntd.0003197

**Published:** 2014-10-02

**Authors:** Brittany Ockenfels, Edwin Michael, Mary Ann McDowell

**Affiliations:** Eck Institute for Global Health, Department of Biological Sciences, University of Notre Dame, Notre Dame, Indiana, United States of America; Liverpool School of Tropical Medicine, United Kingdom

## Abstract

A meta-analysis of the effects of vector saliva on the immune response and progression of vector-transmitted disease, specifically with regard to pathology, infection level, and host cytokine levels was conducted. Infection in the absence or presence of saliva in naïve mice was compared. In addition, infection in mice pre-exposed to uninfected vector saliva was compared to infection in unexposed mice. To control for differences in vector and pathogen species, mouse strain, and experimental design, a random effects model was used to compare the ratio of the natural log of the experimental to the control means of the studies. Saliva was demonstrated to enhance pathology, infection level, and the production of Th2 cytokines (IL-4 and IL-10) in naïve mice. This effect was observed across vector/pathogen pairings, whether natural or unnatural, and with single salivary proteins used as a proxy for whole saliva. Saliva pre-exposure was determined to result in less severe leishmaniasis pathology when compared with unexposed mice infected either in the presence or absence of sand fly saliva. The results of further analyses were not significant, but demonstrated trends toward protection and IFN-γ elevation for pre-exposed mice.

## Author Summary

Arthropod vectors transmit a wide variety of diseases resulting in substantial human morbidity and economic costs worldwide. When hematophagous arthropods blood feed, they release saliva into the host. This saliva elicits a strong immune response and has recently been a focus for vaccine research. There is evidence that the saliva enhances infection in naïve hosts, but that prior exposure to saliva results in less severe infection. This analysis endeavored to determine whether there was a statistically significant enhancement or protective effect with regard to saliva exposure and the progression of disease, and to determine the underlying immune mechanism driving these effects. We found that saliva does indeed enhance infection levels of vector-transmitted pathogens and leishmaniasis pathology in naïve mice and elevates Th2 cytokine levels (IL-4 and IL-10). We also determined that pre-exposure to saliva results in less severe pathology of experimental leishmaniasis in mice. These results are important for vaccine trials and vector control programs, though more studies are needed with regard to pre-exposure.

## Introduction

Vector-borne diseases are a major cause of morbidity and mortality in many areas of the world. In addition to their cost to human health, vector-borne diseases can have a high economic cost primarily affecting impoverished nations and the people with the least resources. While there have been efforts to control or eradicate certain vector-borne diseases, these goals have proved frustratingly elusive and the incidence of some vector-borne infections, such as leishmaniasis, is rising [1]. Emerging and reemerging diseases such as Chikungunya threaten to become major public health concerns. More familiar diseases, like malaria and dengue fever, are infecting new populations due to lapses in vector control programs, human migration and increasing vector habitat due to climate change and other human activities [1]–[8]. Although vaccines have been developed for some vector-borne diseases (e.g. yellow fever,) the vast majority and the most problematic still lack vaccines and viable treatment options. The quest for vaccine development has included assessing the potential protective effect of long-term exposure to insect vector saliva. Results have been mixed at best, and there is some controversy as to whether saliva exacerbates disease or protects against its more severe manifestations.

When an arthropod vector bites a host and transmits a pathogen, it releases some of its own saliva into the bite site as well as the pathogen. It is well established that this saliva is highly immunogenic, containing vasodilatory and immunosuppressive compounds [9]. Perhaps the most studied vectors in this regard have been that of sand fly vectors of leishmaniasis. A landmark study in 1988 by Titus and Ribeiro demonstrated that *Lutzomyia longipalpis* saliva exacerbates *Leishmania major* infection in naïve mice [10]. Many similar studies have followed, consistently demonstrating that naïve animals either infected via sand fly or coinoculation with salivary gland homogenate along with *Leishmania* parasites have generally developed larger, longer lasting lesions than animals inoculated with parasites alone [11]–[25]. Furthermore, these effects appear to be consistent across all sand flies, though the salivary composition differs widely between species. In *Lutzomyia* species, the vasodilatory peptide maxadilan has been implicated in upregulating Th2 cytokines (e.g. IL-4 and IL-10) and down-regulating Th1 cytokines (e.g. IFN- γ) *in vitro* and *in vivo*, presenting a potential mechanism for the observed differences in disease progression [18], [26]–[31]. Belkaid *et al.* further demonstrated that disease enhancement is IL-4 driven, as *Phlebotomus papatasi* saliva did not enhance disease in IL-4 deficient mice. Furthermore, disease enhancement was even greater in IL-12p40 deficient mice [12]. The infection-enhancing effects of saliva, however, have been demonstrated to be negated by prior exposure to uninfected sand fly saliva [11], [13], [18], [24], [32]–[45]. Immunity to the salivary peptides is theorized to elicit a strong Th1 response in the host, which adversely affects *Leishmania* parasites. This effect appears to apply to immunization with uninfected sand fly bites and with individual salivary proteins, though laboratory-colonized sand fly saliva is much more effective than wild-caught in providing protection against disease [11], [13].

Studies assessing the effects of mosquito saliva began soon after those of sand flies, with Bissonnette *et al* and Cross *et al* demonstrating that *Aedes aegypti* saliva inhibits IFN-γ, TNF-α, and IL-2 release from murine cells [46], [47]. As with the sand fly studies, the reports that followed have consistently demonstrated that mosquito saliva from all genera also up-regulates Th2 cytokines and down-regulates Th1 cytokines [46]–[56]. Mosquito saliva has also been shown to increase infectivity of various viruses [57]–[62], as well as enhancing viral replication [63], mortality [64]–[66] and even being necessary for infection [57]. However, there has been some controversy with regard to its effect on malaria, with some studies claiming exacerbation of disease and others claiming no effect or even protection from prior immunization [67]–[70].

Hard ticks are a third group of well-studied arthropod vectors with immunomodulatory saliva. These ticks can take up to two weeks to take a complete bloodmeal, so it is necessary for them to secrete these compounds to avoid rejection from the host. Tick saliva has been demonstrated to inhibit pro-inflammatory (Th1) cytokine production [71]–[79], T cell proliferation [71] and neutrophil activity [80]. Accordingly, it has also been implicated in increasing *Borrelia* and viral infectivity, and immunity against tick saliva may also correspond to decreased effectiveness of the pathogen [81]–[90].

While several review papers on this topic have been published, to date there has not been an analytical comparison of these studies. Here we present a meta-analysis of the effects of vector saliva on disease progression as it applies to three outcomes: pathology, pathogen load, and cytokine levels. Only transient-feeding vectors were included (i.e. sand flies and mosquitoes), as long-term feeding results in a more complicated and not directly comparable interaction. The proportion of mosquito experiments included in each of the analyses varied (22–52% for pathogen load, 25–37.5% for cytokine levels, and 0% for pathology) due to the limited number of published studies including these parameters. Also as a result of paucity, human studies and research on trypanosomes and their vectors were also excluded. For comparability, only *in vivo* infection, as opposed to macrophage and other in *vitro* cell studies, and quantification by ELISA and PCR were used for the cytokine evaluation. Furthermore, only IFN-γ, IL-4, and IL-10 were included, as they were the most often studied.

Experiments were placed into two groups: naïve animals exposed to saliva during infection compared with a control group exposed to only pathogens, and animals pre-exposed to saliva before infection compared with a control group of naïve animals exposed to saliva only during infection. A third group, pre-exposed animals compared with those that were needle inoculated and not exposed to saliva at all, was included in the leishmaniasis pathology evaluation. Other than expanding our knowledge of the biology of infection, the results of the analyses concerning the first group could have ramifications for vector control programs and vaccine studies. If control programs are allowed to lapse, newly naïve populations could end up with more severe disease. As for vaccine trials, it would be important to test against vector-borne infection as opposed to needle inoculation. This has been a problem especially with vaccines against leishmaniasis; they may work for needle-inoculated mice but fail to protect against infection via sand fly [91], [92]. The second group mimics natural conditions for endemic populations and naïve ones such as travelers and deployed military service members. It is important to understand potential elevated risks in these populations, as well as potential for vaccine studies. The third group assesses whether immunity induced by saliva pre-exposure just negates the exacerbative effect of saliva, or if there is an added protective effect. While there are certainly limits to this type of analysis, it can be very useful in determining whether observed trends across the published literature are statistically significant effects.

## Methods

### Data Sources and Extraction

For consistency and comparability, this analysis included only murine studies concerning transient-feeding vectors. A thorough literature search was performed using Pubmed (http://www.ncbi.nlm.nih.gov/pubmed/) for papers published until May 2013. Search terms combined vector saliva, immune response, and specific vectors and diseases such as leishmaniasis, malaria, dengue, sand fly saliva, and mosquito saliva. Other papers were found using the references in previously located articles. Criteria for inclusion were studies using wild-type mouse strains (as opposed to certain immunodeficient) that contained information on one of three outcomes: pathology (leishmaniasis papers only), cytokine levels in vivo (ELISA or PCR), and infection level (parasite or viral load in tissues or parasitemia/viremia). Due to the constraints inherent in a meta-analysis, we limited our focus on pathology to studies evaluating leishmaniasis. The statistical analyses required a certain number of data points and we were unable to find enough studies assessing other pathogens to make comparisons on their own and we could not combine the studies with leishmaniasis pathology studies because the types of assays were not comparable (e.g. lesion size compared to mortality analysis). Similarly, flow cytometric analysis of cytokine expression was excluded because there was not enough conformity across studies in the experimental set up, cells assessed, and gating strategies to be controlled properly.

The Studies were organized into three broad categories of experiments for analysis:

1. Studies comparing a control group of naïve mice inoculated with the pathogen only to an experimental group of naïve mice infected with the pathogen either by vector feeding or by co-inoculation with the pathogen and a vector salivary gland extract (Saliva vs Control).
2. Studies comparing a control group of naïve mice infected with the pathogen by vector feeding or by co-inoculation with the pathogen and saliva to an experimental group of mice pre-exposed to vector saliva then infected with the pathogen along with saliva (Pre-exposed vs Saliva).
3. Studies comparing a control group of naïve mice inoculated with the pathogen only to an experimental group of mice pre-exposed to vector saliva then infected with the pathogen along with saliva (Pre-exposed vs Control).

Cytokines were divided into IFN-γ, IL-4, and IL-10 groups, as these proteins were most commonly measured. Mean values and standard deviations of the mean for each outcome were extracted from either data reported in the papers or from figures using the “grabit” function in MATLAB (Mathworks). When standard errors were reported, they were converted to standard deviations with the formula SE = SD/√N. When no standard deviation or error was reported, standard deviation was calculated as 1/N. The mean was taken for multiple measurements over time.

### Data Analysis

The data were analyzed with the metafor package in R (r-project.org). A random effects model was used as there is considerable variation in both mouse strain and vector and pathogen species. The natural log of the ratio of the experimental mean to the control mean was taken [Yi = ln(Xe/Xc)]. Using a ratio allowed us to directly compare studies and provided a means of controlling for differences in experimental design. Variance was calculated by the formula Vi = [SDe^2^/(Ne*Xe^2^)]+[SDc^2^/(Nc*Xc^2^)]. The code used in R was as follows:

dat1<-read.csv(“[file name]”, header = TRUE)

res1<- rma(Yi,Vi, data = dat1)

summary(res1)

forest(res1, slab = paste(dat1$Author, dat1$Year, sep = “,”))

This code sequence provided a summary of the analysis (most importantly overall effect and p value) and a forest plot of the data for each group.

## Results

### Infection Level

**Figure 1:**
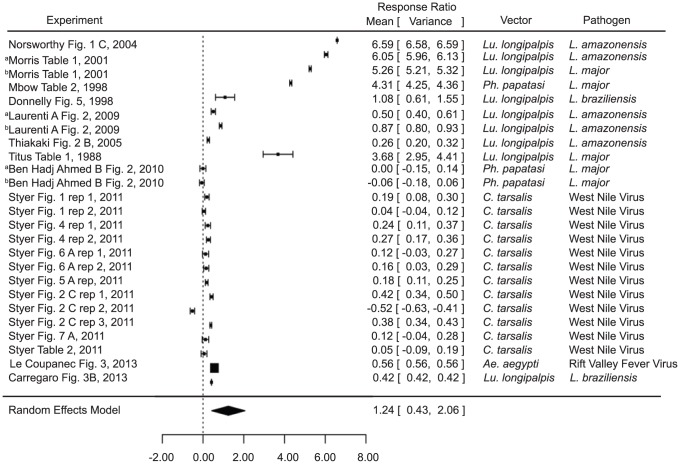
Forest plots of the relationship of vector saliva and infection level in naïve mice (Category 1). Symbols represent the mean response ratio of the individual studies (squares) and of the entire analysis (diamond) using a Random Effects Model; the size of the square is proportional to the weight of an individual study. Error bars represent 95% Confidence Interval (CI). Squares to the right of the dotted line indicate larger measurements in the experimental (saliva) group, while those on the left indicate larger measurements in the control group. Those that cross the center indicate no significant difference.

The infection level analysis combined measurements of parasite and viral load in tissues and parasitemia or viremia. Various sand fly and mosquito vectors were included, as were various pathogen species (namely *Leishmania* species, *Plasmodium* species, and West Nile virus) (Table S1). Infection of naïve mice in the presence of vector saliva was found to significantly increase infection level (estimate 1.2440, p value 0.0029) compared to pathogen alone (Fig. 1). These results are broadly applicable, considering the variation in vectors (*Lu. longipalpis*, *Ph. papatasi*, *Culex tarsalis*, and *Ae. aegypti*) and pathogens (*L. amazonensis*, *L. major*, *L. braziliensis*, West Nile Virus, and Rift Valley Fever Virus).

Pre-exposure to saliva, however, was not demonstrated to significantly decrease infection level across all vectors and pathogens (estimate −0.6266, p value 0.0868, Table S2) or even across just the sand fly vectors and leishmaniases (estimate −0.8063, p value 0.0865, Table S2). The general trend, however, did indicate protection. There was unfortunately not enough information available to perform an analysis of the third group, that of pre-exposed mice compared with control mice infected without saliva.

In some of the studies, a salivary protein was used as a proxy for saliva as a whole (maxadilan [18] and rLMJ11 [38]). The analysis was conducted both including these studies (Fig. 1; Table S2) and excluding them (Table S2), and the results were not significantly different from each other. Although saliva is a complex cocktail of proteins and the protocols utilized for vaccination utilize greater amounts of a single protein than is found in salivary extracts, this result indicates that maxadilan and LmJ11 are both likely major factors in saliva's immunogenic properties, and that they alone have nearly the same effect as the entire salivary gland homogenate.

### Pathology

**Figure 2:**
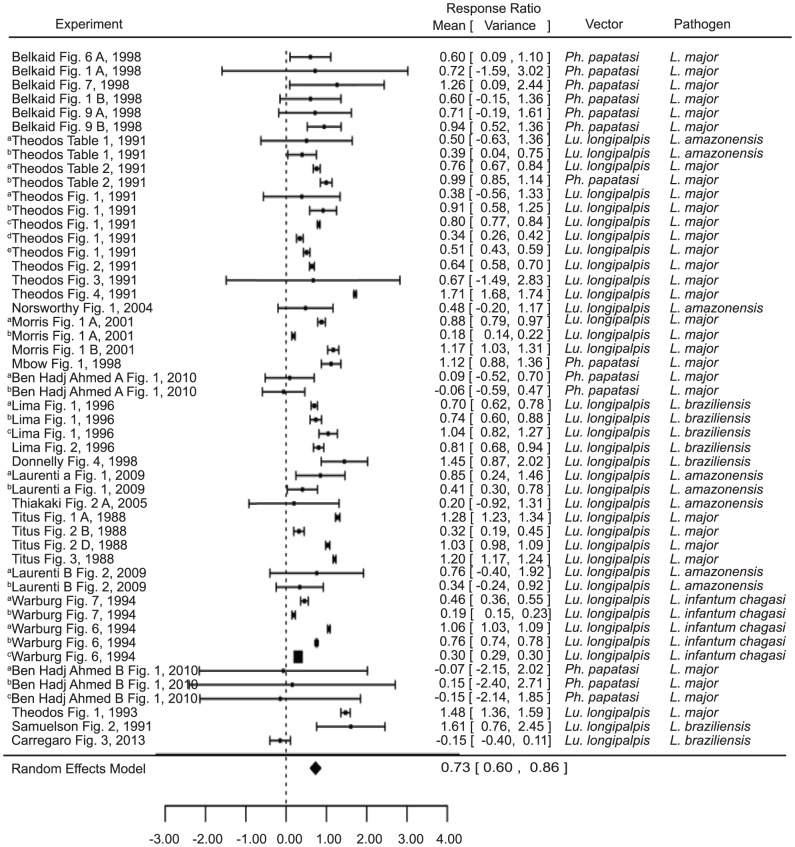
Forest plots of the relationship of exposure to vector saliva and *Leishmania* lesion size in naïve mice (Category 1). Symbols represent the mean response ratio of the individual studies (squares) and of the entire analysis (diamond) using a Random Effects Model; the size of the square is proportional to the weight of an individual study. Error bars represent 95% Confidence Interval (CI). Squares to the right of the dotted line indicate larger measurements in the experimental (saliva) group, while those on the left indicate larger measurements in the control group. Those that cross the center indicate no significant difference.

Due to the low number of studies concerning other aspects of pathology, here pathology is synonymous with the size of *Leishmania* induced lesions. Though all of the studies in this analysis concerned sand flies and *Leishmania*, they varied considerably with regard to mouse strain, sand fly species, *Leishmania* species, and experimental design (e.g. infected ear or footpad, experimental group infection by vector feeding or inoculation, amount and times of pre-exposure, etc). Consistent with the infection level results, naïve mice infected in the presence of saliva had significantly larger lesions than those in the control group (estimate 0.319, p value<0.001) (Fig. 2). These results were consistent regardless of whether the saliva came from the natural vector or another species of sand fly (natural vector estimate 0.6183, p value<0.0001; other vector estimate 0.7837, p value<0.0001; Table S2), or even whether the vector was of the natural genus (natural genus estimate = 0.6388, p = <0.0001, other genus estimate 0.8644, p value<0.0001) (Table S2). Saliva also appeared to increase the duration of the lesions, though this factor was not included in the analysis due to inconsistencies in the lengths and intervals of time measured between studies.

**Figure 3:**
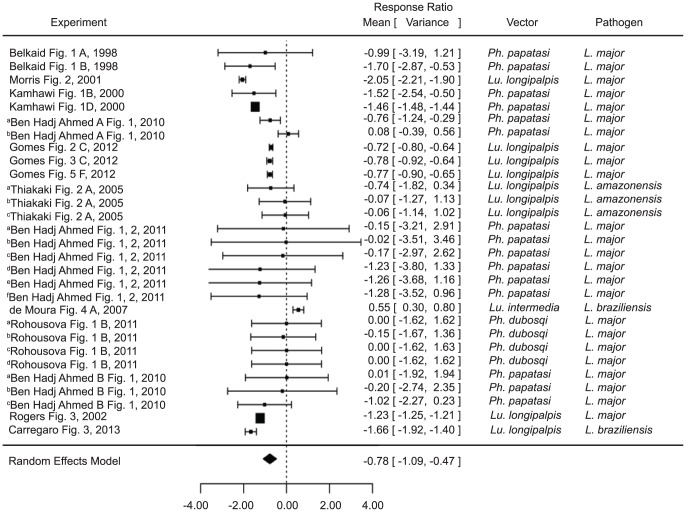
Forest plots of the relationship of exposure to vector saliva before infection and *Leishmania* lesion size (Category 2). Symbols represent the mean response ratio of the individual studies (squares) and of the entire analysis (diamond) using a Random Effects Model; the size of the square is proportional to the weight of an individual study. Error bars represent 95% Confidence Interval (CI). Squares to the right of the dotted line indicate larger measurements in the experimental (pre-exposed) group, while those on the left indicate larger measurements in the control group. Those that cross the center indicate no significant difference.

Pre-exposure to saliva, however, was shown to significantly decrease lesion size (estimate −0.7781, p value<0.0001) when compared with naïve mice infected in the presence of the same saliva (Fig. 3). Interestingly, the only study to show the opposite [35] was also the only study conducted on the natural pairing of *L. braziliensis* and *L. intermedia*. However, the overall results again remained significant regardless of whether the saliva came from the natural vector or even the natural genus (natural vector estimate −.5839, p value 0.0074, other vector estimate −1.0174, p value<0.0001, natural genus estimate −0.6909, p value 0.0008, other genus estimate −09326, p value 0.0010) (Table S2). It is noteworthy that two of the experiments (^b^Thiakaki Fig. 2A and ^c^Thiakaki Fig. 2A [24]) included, trend more toward enhancement, though not statistically significant. The mice in these studies were pre-exposed to *Ph. papatasi* and *Ph. sergenti* saliva, respectively, and subsequently exposed to *Lu. longipalpis* saliva upon infection. The third experiment by the same authors(^a^Thiakaki Fig. 2A [24]), where mice were pre-exposed to *Lu. longipalpis* saliva, demonstrated protection. These results imply that the protection gained by prior exposure may be somewhat species (or at least genus)- specific.

**Figure 4:**
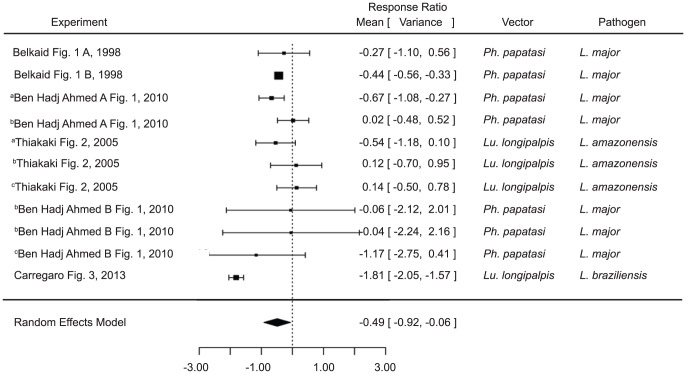
Forest plots comparing pre-exposure to vector saliva to control groups infected in the absence of saliva on leishmaniasis pathology (Category 3). Symbols represent the mean response ratio of the individual studies (squares) and of the entire analysis (diamond) using a Random Effects Model; the size of the square is proportional to the weight of an individual study. Error bars represent 95% Confidence Interval (CI). Squares to the right of the dotted line indicate larger measurements in the experimental (pre-exposed) group, while those on the left indicate larger measurements in the control group. Those that cross the center indicate no significant difference.

When compared with a control group infected without any saliva at all, mice pre-exposed to saliva developed smaller lesions (estimate −0.4889, p value 0.0254) (Fig. 4). As with the infection level studies, the analysis did not vary by excluding the studies using only maxadilan or rLMJ11 (Table S2).

### Cytokines

**Figure 5:**
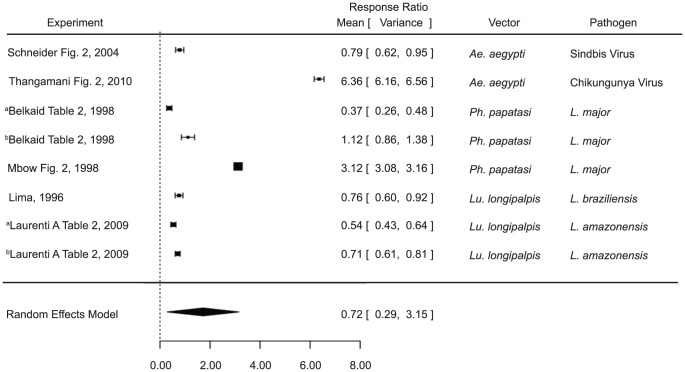
Forest plots of the relationship of vector saliva and IL-4 levels in naïve mice. Symbols represent the mean response ratio of the individual studies (squares) and of the entire analysis (diamond) using a Random Effects Model; the size of the square is proportional to the weight of an individual study. Error bars represent 95% Confidence Interval (CI). Squares to the right of the dotted line indicate larger measurements in the experimental (saliva) group, while those on the left indicate larger measurements in the control group. Those that cross the center indicate no significant difference.

Studies included in the cytokine analysis were those that measured IFN-γ, IL-4, or IL-10 by either ELISA or PCR. Other cytokines and those measured via flow cytometry were excluded for consistency and due to low numbers and only measurements from *in vivo* infections were included. IFN-γ analysis of pre-exposed versus naïve mice (n = 5), as well as, of naïve mice infected in the presence versus absence of saliva (n = 9) were inconclusive (Table S2). Though general trends were observed, they were not significant (IFN-γ levels were lower in naïve mice exposed to saliva than in control mice and higher in pre-exposed mice than in the control group, Table S2). Naïve mice exposed to saliva during infection, however, had significantly higher IL-4 levels than control mice exposed only to the pathogen (estimate 1.7196, p value 0.0185) (Fig. 5).

**Figure 6:**
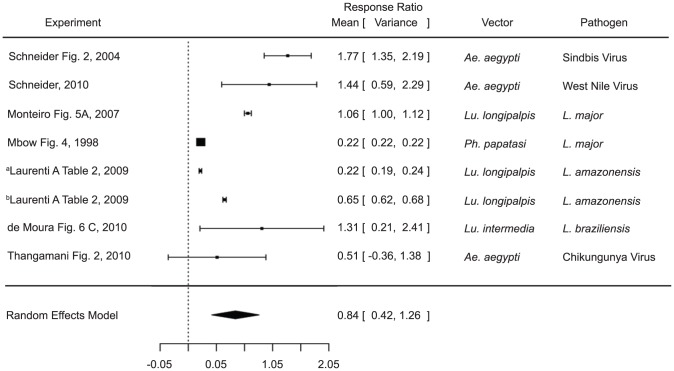
Forest plots of the relationship of vector saliva and IL-10 levels in naïve mice. Symbols represent the mean response ratio of the individual studies (squares) and of the entire analysis (diamond) using a Random Effects Model; the size of the square is proportional to the weight of an individual study. Error bars represent 95% Confidence Interval (CI). Squares to the right of the dotted line indicate larger measurements in the experimental (saliva) group, while those on the left indicate larger measurements in the control group. Those that cross the center indicate no significant difference.

Likewise IL-10 levels were shown to be significantly higher in naïve mice exposed to saliva during infection (estimate 0.8398, p value<0.001) (Fig. 6). Unfortunately there were not enough measurements of IL-4 or IL-10 in mice pre-exposed to saliva versus naïve mice to conduct an analysis. Both of the cytokine findings were consistent across studies using both mosquito and sand fly vectors and various pathogens (parasitic and viral), suggesting a common mechanism of disease enhancement in the saliva of diverse vectors.

## Discussion

Here we performed a meta-analysis of available data concerning the effects of vector saliva on host immunity. While a vast amount of heterogeneity existed between studies, the use of a ratio allowed us to control for the variability. Overall, our study indicates that saliva enhances infection in naïve mice. Pathogen levels in host blood and tissues are consistently higher in those mice exposed to saliva during infection and this effect holds true for both sand fly and mosquito vectors and for different pathogen species. As such, one would imagine that these results could be extended to other transient-feeding vectors as well, and indeed that has been demonstrated to be the case with *Glossina morsitans morsitans*/*Trypanosoma brucei brucei* [93], [94] and *Rhodnius prolixus*/*Trypanosoma cruzi* [95]. Both of these studies report higher parasitemia in naïve mice infected in the presence of saliva. More studies need to be performed on these and other vectors, however, to see if the findings are truly consistent. In addition to its effects on infection level, vector saliva also influences leishmaniasis pathology. Here we demonstrated that sand fly saliva enhances *Leishmania*-induced lesion size. Furthermore, higher levels of morbidity and mortality in mice infected with West Nile virus in the presence of mosquito saliva have been reported [61], [64]–[66]. Thus, the higher infection levels that result from saliva exposure have a demonstrable effect on the disease pathology.

An important consideration in this analysis was whether the vector/pathogen pairing was natural or unnatural. Several sand fly/*Leishmania* studies used an unnatural combination of *Lu. longipalpis* saliva with *L. major*, where the natural vector is one of several *Phlebotomus* species [10], [18], [22], [23], [38], [41], [96]. Likewise there have been studies using *Lu. longipalpis* saliva paired with *L. amazonensis* or *e. braziliensis* [14]–[16], [18], [19], [21], [22], [24], [97], [98]. While the genus is correct, these *Leishmania species* are naturally transmitted by different sand fly species [99]. These studies have the potential be both helpful and misleading. What is true for an unnatural pairing may not hold for a natural combination and thus the results may have little practical application. On the other hand, if the effects are similar regardless of the pairing, there may be potential for a more comprehensive vaccine, or at the least important implications for travelers already exposed to different sand fly species. We found that including or excluding the unnatural pairings made no difference in the overall results of the analyses, and that both natural and unnatural pairings (species and genus) generally demonstrated the same results in all categories.

A proposed mechanism for the salivary enhancement of infection has been the up-regulation of host Th2 cytokines. Indeed our analysis demonstrates a marked increase in IL-4 and IL-10 levels in groups exposed to saliva, both sand fly and mosquito, suggesting a strong Th2 response. These results, taken with the lack of enhancement in IL-4 deficient mice [12], strongly imply that the proposed Th2 driven mechanism is in fact correct. *In vivo*, cytokines function in a milieu of other cytokines and factors and it is the relative balance (or ratio) or these proteins that set the tone of a particular immune response. Whether Th1 cytokines are were regulated in response to saliva exposure is another question we investigated. While IFN- γ levels were generally lower in mice exposed to saliva, the results were not significant. However, upon further examination of the data, the only study to report the opposite also contained the only unnatural vector/pathogen pairing ([96], *Lu. longipalpis/L. major*). Eliminating this study lowered the p value, but not enough that the results were significant.

The potential for vaccines developed from vector saliva has been an important research topic in recent years. Therefore, a major aim of this study was to determine whether pre-exposure to vector saliva results in less severe infection. In the infection level analysis, while the trend was toward lower levels in pre-exposed mice, the results were not significant and therefore inconclusive. However, the leishmaniasis pathology analysis demonstrated less severe lesions in pre-exposed mice, and this result holds true even when compared with mice unexposed to saliva even during infection. Therefore, with respect to leishmaniasis pathology, pre-exposure does not just negate the infection-enhancing effects of saliva in naïve mice, it actually confers a significant protective effect compared to infection in the absence of saliva. It is interesting to note, however, that while pre-exposure to *Lu. intermedia* saliva does decrease infection level, it appears to have the opposite effect on lesion size [35]. More studies are necessary to investigate this phenomenon.

While a comprehensive cytokine analysis would be extremely informative with regard to the mechanism of the demonstrated protective effect, unfortunately there were not enough pertinent studies to conduct an analysis on IL-4 or IL-10 levels, and the IFN- γ analysis results were inconclusive. Kamhawi *et al.* found little change in the level of IL-4 producing cells in pre-exposed mice compared with naïve mice [39], though IFN- γ levels were elevated in pre-exposed mice. Interestingly, all of the studies reported much higher IFN- γ levels in pre-exposed mice except for one using a natural pairing of *Ph. papatasi/L. major* in BALB/c mice [12]. The same pairing with C57BL/6 mice indicated elevated IFN- γ in pre-exposed mice. This study is the only incidence in the analyses where mouse strain makes a difference, but it illustrates that while these results may be true for some mouse strains and vector/pathogen combinations, they may not be true for other strains or indeed other animals or humans.

Not surprisingly, studies assessing human immune responses to insect bites in disease settings are few and do not present a unifying theme for all vector-transmitted diseases or for a single disease or vector. Some studies suggest that saliva exposure skews the human immune response toward Th2-type immunity [100]–[103] and others suggest a more mixed response [104]–[106].

This meta-analysis has demonstrated conclusively the infection-enhancing effect of transient-feeding vector saliva in murine models of infection and the Th2 driven mechanism behind it; however, more studies need to be conducted on the effects of pre-exposure. A significant protective effect exists with regard to sand fly saliva and leishmaniases, but the mechanism still needs to be clarified. More cytokine studies are needed, as well as additional studies with other. Overall, the vaccine potential of saliva needs to be further investigated. There are many important considerations in the potential development of vaccines, not least that humans may be affected very differently than specific mouse strains, saliva differs widely between vectors, and immunity to saliva has only been demonstrated to result in less severe disease, not prevent infection entirely. Indeed, while the human response to vector saliva has been demonstrated to be similar to the murine one in that saliva enhances infection in naïve human cells [107]–[109], the effects of pre-exposure have been more controversial and appear to be more complicated than in mice [100]–[102], [105], [106].

This study, perhaps most importantly, emphasizes the importance of maintaining vector control programs once started. If allowed to lapse, not only will the protective immunity be lost when vector populations rebuild, but disease may be much more severe in newly naïve populations.

## Supporting Information

Table S1 **Metadata associated with each experiment included in analysis.** Excel file containing metadata of each experiment. Metadata includes, figure, vector, saliva component, pathogen, natural/unnatural pairing, mouse strain.
(XLSX)

Table S2 **Statistical analysis of each test.** Response ration and P-values for each test are reported.
(XLSX)
